# Diagnosing and tracking depression based on eye movement in response to virtual reality

**DOI:** 10.3389/fpsyt.2024.1280935

**Published:** 2024-02-05

**Authors:** Zhiguo Zheng, Lijuan Liang, Xiong Luo, Jie Chen, Meirong Lin, Guanjun Wang, Chenyang Xue

**Affiliations:** ^1^ School of Information and Communication Engineering, Hainan University, Haikou, China; ^2^ School of Information Engineering, Hainan Vocational University of Science and Technology, Haikou, China; ^3^ The First Affiliated Hospital of Hainan Medical University, Haikou, China; ^4^ Department of Psychology, University of Chinese Academy of Sciences, Beijing, China; ^5^ School of Electronic Science and Technology, Hainan University, Haikou, China

**Keywords:** depression, diagnose, XGBoost, MLP, CCBT

## Abstract

**Introduction:**

Depression is a prevalent mental illness that is primarily diagnosed using psychological and behavioral assessments. However, these assessments lack objective and quantitative indices, making rapid and objective detection challenging. In this study, we propose a novel method for depression detection based on eye movement data captured in response to virtual reality (VR).

**Methods:**

Eye movement data was collected and used to establish high-performance classification and prediction models. Four machine learning algorithms, namely eXtreme Gradient Boosting (XGBoost), multilayer perceptron (MLP), Support Vector Machine (SVM), and Random Forest, were employed. The models were evaluated using five-fold cross-validation, and performance metrics including accuracy, precision, recall, area under the curve (AUC), and F1-score were assessed. The predicted error for the Patient Health Questionnaire-9 (PHQ-9) score was also determined.

**Results:**

The XGBoost model achieved a mean accuracy of 76%, precision of 94%, recall of 73%, and AUC of 82%, with an F1-score of 78%. The MLP model achieved a classification accuracy of 86%, precision of 96%, recall of 91%, and AUC of 86%, with an F1-score of 92%. The predicted error for the PHQ-9 score ranged from -0.6 to 0.6.To investigate the role of computerized cognitive behavioral therapy (CCBT) in treating depression, participants were divided into intervention and control groups. The intervention group received CCBT, while the control group received no treatment. After five CCBT sessions, significant changes were observed in the eye movement indices of fixation and saccade, as well as in the PHQ-9 scores. These two indices played significant roles in the predictive model, indicating their potential as biomarkers for detecting depression symptoms.

**Discussion:**

The results suggest that eye movement indices obtained using a VR eye tracker can serve as useful biomarkers for detecting depression symptoms. Specifically, the fixation and saccade indices showed promise in predicting depression. Furthermore, CCBT demonstrated effectiveness in treating depression, as evidenced by the observed changes in eye movement indices and PHQ-9 scores. In conclusion, this study presents a novel approach for depression detection using eye movement data captured in VR. The findings highlight the potential of eye movement indices as biomarkers and underscore the effectiveness of CCBT in treating depression.

## 1 Introduction

The “World Mental Health Report 2022” highlights a concerning trend: the number of people with mental illness has surpassed one billion worldwide. The coronavirus disease 2019 pandemic has exacerbated this issue, with a 25% increase in the number of people experiencing anxiety and depression in the first year of the pandemic Organization et al. (1). Depression alone is responsible for over 800,000 deaths annually Cai et al. (2), and its prevalence has increased by 18% over the past decade Mahato and Paul (3).

Currently, the clinical diagnosis of depression primarily relies on psychological behavioral scales, such as the Hamilton Depression Scale Hamilton (4), Beck Depression Inventory Beck et al. (5), and Self-Rating Depression Scale Zung (6). However, these methods are limited by subjectivity and lack objective and quantitative evaluation criteria, increasing the risk of various subjective biases Sung et al. (7). Moreover, completing these questionnaires is time consuming, with each form requiring at least 30 minutes. Therefore, finding an objective and efficient diagnostic method is an urgent problem that must be addressed Strawbridge et al. (8); de Aguiar Neto and Rosa (9); Tavakolizadeh et al. (10); Chojnowska et al. (11).

The phrase “the eyes are the windows to the soul” holds true in psychology; changes in pupil size are related to psychological states, reflecting an individual’s higher cognitive processes Hess and Polt (12). Pupil size measurements have been used to evaluate stress Yamanaka and Kawakami (13). Eye movement indices such as fixation duration, saccade count, and pupil size are direct reflections of brain information processing and can quantitatively characterize emotional perception. Previous studies have established a connection between eye movement indices and depression. Free-viewing, fixation stability, and smooth pursuit tests were conducted, resulting in 35 eye movement measurements. The results revealed that in the free viewing test, individuals with Major Depressive Disorder (MDD) exhibited significantly shorter scanpath length. In the smooth pursuit test, the duration of saccades was significantly shorter, and the peak saccade velocity was significantly lower in individuals with MDD Takahashi et al. (14).The prosaccade task and the antisaccade task were performed using iViewX RED 500 eye-tracking instruments. The results showed significant differences between the depression group and the control group in the antisaccade task, specifically in the correct rate (t = 3.219, p = 0.002) and mean velocity (F = 3.253, *p<* 0.05) Gao et al. (15). Eyelink 1000 recorder was used to collect eye movement data. Features related to fixation, saccades, pupil size, and depression preference were extracted from the data. Five classifiers, namely k-Nearest-Neighbor (kNN), Naïve Bayes (NB), Logistic Regression (LR), Support Vector Machine (SVM), and Random Forest (RF), were employed to classify a group of students into depressed and normal categories. By utilizing eye movement features during free viewing tasks, an accuracy of 80.1% was achieved using Random Forest to differentiate between depressed and non-depressed subjects Li et al. (16).Eye movement metrics can also be combined with other indicators. Facial expressions and eye movement data were integrated, and Classification Accuracy using cross-validation (within-study replication) achieved a level of 79% (sensitivity 76%, specificity 82%).Stolicyn et al. (17). Eye movement data and resting-state EEG signals were combined, and an ensemble method was employed. The study utilized free viewing eye tracking and resting-state EEG data, and the results demonstrated that the combined approach, known as CBEM, achieved accuracies of 82.5%.Zhu et al. (18).Eye movement indices can effectively be used to distinguish depression from other mental illnesses and serve as biomarkers to aid in the diagnosis and evaluation of depression Bae et al. (19); Pan et al. (20); Carvalho et al. (21); Wen et al. (22); Wang et al. (23); Carvalho et al. (24). These findings are supported by several studies.

**Figure 1:**
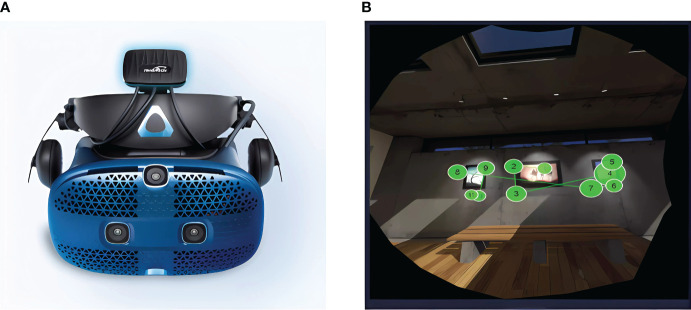
Selected techniques for depression and the eye movement trajectory: **(A)** VR eye tracker, **(B)** eye movement trajectory.

An eye tracker is an essential instrument for measuring eye movement indices and trajectories. We used a virtual reality (VR) eye tracking system comprised of an aSee VR (Beijing 7invensun Technology Co., Ltd., Beijing, China) and HTC Vive Pro (HTC Corporation, Taoyuan City, Taiwan), as shown in **Figure 1**. This eye tracking system differs from commonly used series such as the EyeLink or Tobii. This VR eye tracking system provides a more immersive and realistic experience because of its ability to create rich virtual environments. Moreover, psychotherapy and interventions based on VR have shown promise for the treatment of patients with anxiety and depression Li et al. (25); Jiede et al. (26); Suwanjatuporn and Chintakovid (27); Torous et al. (28).

Machine learning algorithms can analyze data, thereby enabling the prediction and classification of practical problems Islam et al. (29); Chiong et al. (30); AlSagri and Ykhlef (31); Narayanrao and Kumari (32). Recently, the use of physiological indices such as speech Herniman et al. (33); Cohen et al. (34); Rapcan et al. (35) and electroencephalogram signals de Aguiar Neto and Rosa (9); Keren et al. (36); Acharya et al. (37) has facilitated the collection and analysis of data for the purpose of diagnosing depression and distinguishing individuals with depression from healthy individuals. These are fast, effective, and efficient auxiliary methods for detecting depression.

Computerized cognitive behavioral therapy (CCBT) is an online tool that enables patients to receive professional cognitive behavior therapy and has gained significant popularity in recent years Wickersham et al. (38); Wright et al. (39). CCBT is not bound by time or location, effectively solving the issue of insufficient medical resources Wright et al. (40); Pfeiffer et al. (41).

Based on the reviewed literature, we aimed to utilize machine learning methods to detect depression by analyzing eye movement data obtained from VR scenes. In addition, this study investigated the effectiveness of CCBT in treating depression.

## 2 Materials and methods

### 2.1 Participant information

The Participants were recruited from Hainan Medical University and assessed using the PHQ-9 scale Kroenke et al. (42) based on the inclusion and exclusion criteria. The PHQ-9 scale, developed by Kroenke et al. in 2001, is based on diagnostic criteria for depressive episodes. A total of 167 participants with PHQ-9 scores of 5 or higher, indicating significant depressive symptoms, were selected for the study. Subsequently, 60 participants were selected based on the inclusion criteria and divided into a depression intervention group and a depression control group, with 30 participants in each group. In addition, 30 students were selected as the healthy control group from 124 participants who scored 4 points or lower on the PHQ-9 scale, indicating no or minimal depressive symptoms, according to the inclusion criteria. The inclusion and exclusion criteria are presented in **Table 1**.

**Table 1:** The inclusion and exclusion criteria of eye tracker experiments for depression detection.

	Participant with depressive emotion	healthy controls
Inclusion criteria	1 Between the age of 16-25 years old, male or female 2 All participants are university students 3 The PHQ-9 score of the subject was greater than or equal to 5. 4 No psychotropic drug treatment having been performed in the last two weeks	1 Between the age of 16-25 years old, male or female 2 All participant are university students 3 No history of mental illness 4 PHQ-9 score of subject was lower than 4
exclusion criteria	1 History of significant physical illness 2 have suicidal tendency 3 have bipolar affective disorder and psychotic symptoms	1 Individual or family with mental illness 2 with other serve physical illness 3 Were dependent on alcohol or psychotropic drugs in the past year

In the study, the participants were categorized based on their PHQ-9 scores. Among the participants, 13 individuals were classified as having mild depression (scores of 5-9), 33 individuals were classified as having moderate depression (scores of 10-14), 8 individuals were classified as having moderately severe depression (scores of 15-19), and 6 individuals were classified as having severe depression (scores of 20-27).During the experiment, there was participant attrition, and a total of 69 individuals completed the experiment. Among them, 24 individuals were in the healthy group, and 45 individuals were in the depressive emotion group. This study was reviewed and approved by the Ethics Committee of The First Affiliated Hospital of Hainan Medical University. All participants provided written informed consent.

### 2.2 Experimental design

**Figure 2:**
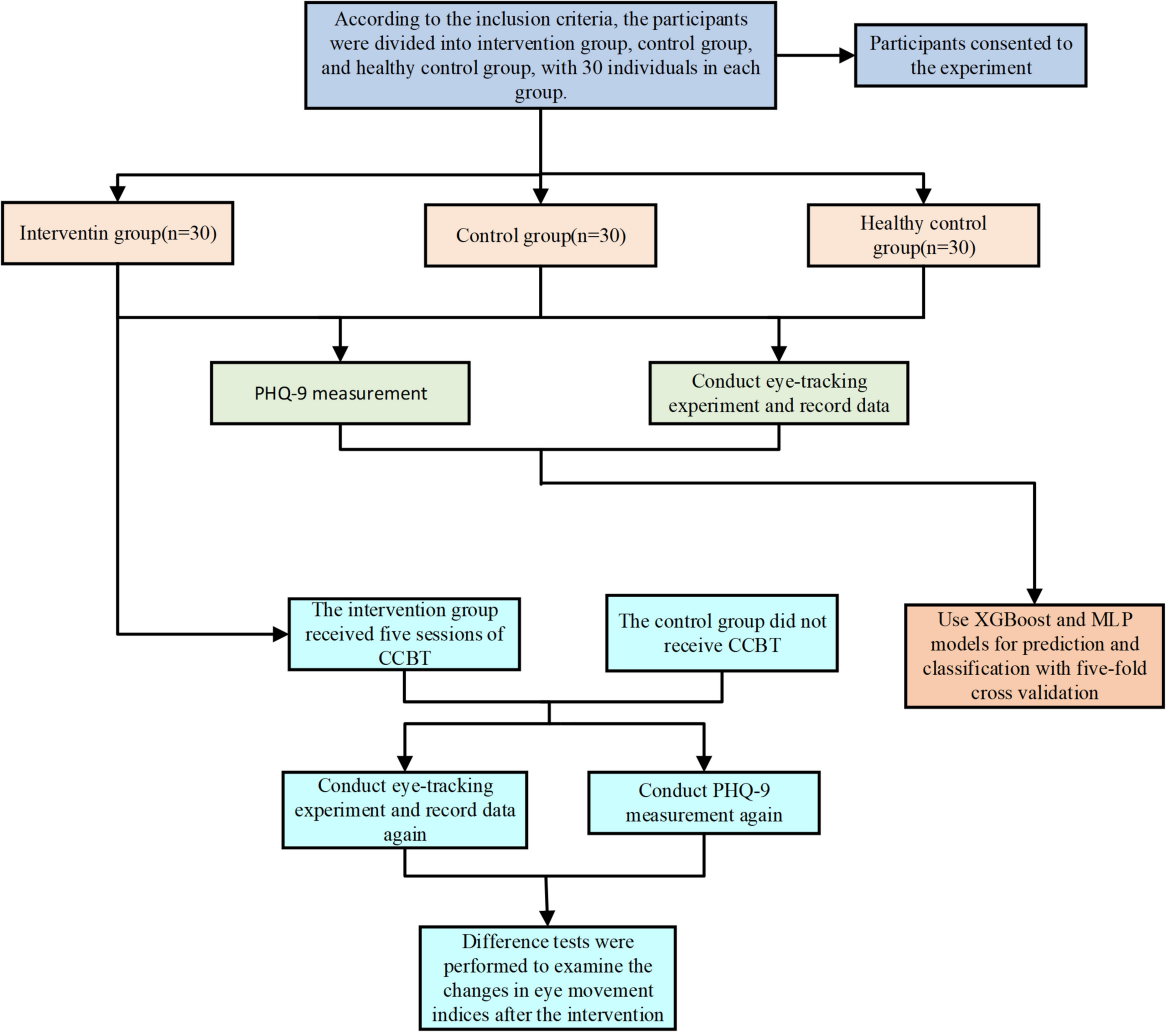
Experimental procedure.

The participants were divided into three groups: depression intervention, depression control, and healthy control, with 30 individuals in each group. Next, the participants completed the PHQ-9 and their eye movement data was collected using the HTC Vive Eye Pro eye-tracking device. The data was then processed, classified, and used to predict PHQ-9 scores using XGBoost Chen and Guestrin (43) and MLP Fatima et al. (44) models. The performance of the two models was compared. After completing five sessions of group-based CCBT, we readministered the PHQ-9 and repeated the eye movement experiment in the intervention group. The results were compared with those obtained before the CCBT intervention to assess the changes in PHQ-9 scores and eye movement data. The experimental flowchart is shown in **Figure 2**.

### 2.3 Feature extraction

The definition of eye movement indices can be found in reference Holmqvist et al. (45). For this experiment, eight eye-movement indices were extracted as input parameters for model training using a VR eye tracker. These indices included fixation count, fixation duration, mean fixation duration, saccade count, saccade amplitude, mean saccade amplitude, and the average pupil sizes of both eyes.

### 2.4 Model introduction

#### 2.4.1 XGBoost model

In this study, we analyzed the collected data separately using the XGBoost and MLP models. The XGBoost model was used for classification and prediction. XGBoost is an upgraded version of the gradient-boosted decision tree algorithm Wang et al. (46) that uses a gradient-boosting algorithm, similar to the boosting method used in ensemble learning. Ensemble learning algorithms such as XGBoost construct multiple weak learners and aggregate the modeling results of all weak learners to obtain better classification or regression performance than that of a single model. XGBoost is known for its efficiency, scalability, and accuracy, making it a popular choice for machine learning applications, including those related to mental health.

The modeling process involves building a tree to form an evaluator that is gradually iterated. During each iteration, the evaluator focuses on easily misclassified samples and gradually integrates strong evaluators.

**Figure 3:**
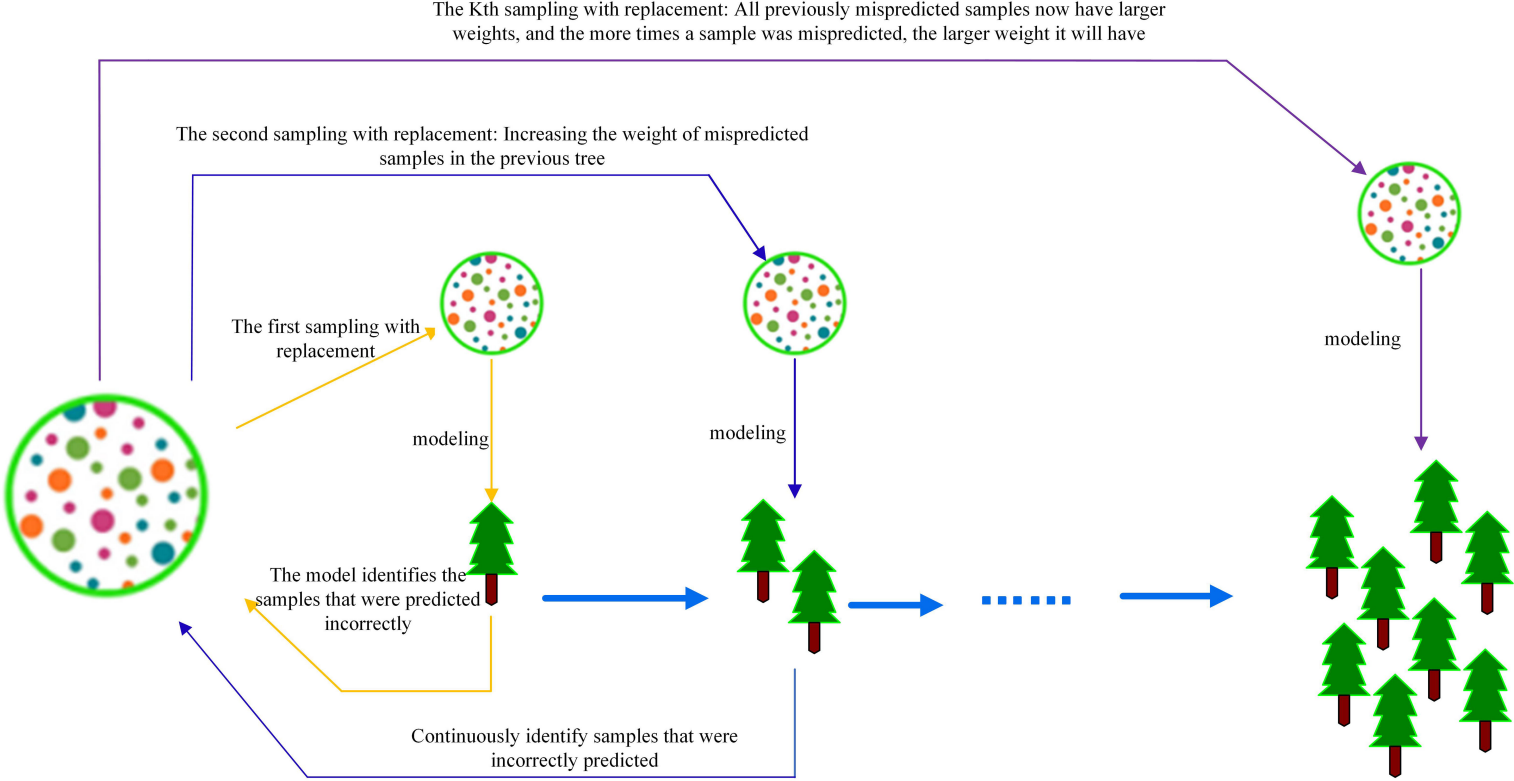
The process of building an XGBoost model.

This iterative process enables the XGBoost model to improve its performance over time, leading to increasingly accurate predictions. **Figure 3** illustrates the modeling process of the XGBoost algorithm.

Multiple decision trees were used during the process of building the XGBoost model, and the regression result of the ensemble model is the sum of the predicted scores for all trees. If K decision trees exist in the ensemble model, the predicted result of the entire model for sample I can be expressed as follows (**Equation 1**):


(1) $${\hat{y_{i}}}^{\left(k\right)}=\sum_{k}^{K}f_{k}\left(x_{i}\right)$$

The objective function of the XGBoost model can be expressed as a combination of the loss function and model complexity as follows (**Equation 2**):


(2) $$obj=-\frac{1}{2}\sum_{j=1}^{T}\frac{G_{j}^{2}}{H_{j}+\lambda }+\gamma T$$

#### 2.4.2 Multilayer perceptron model

**Figure 4:**
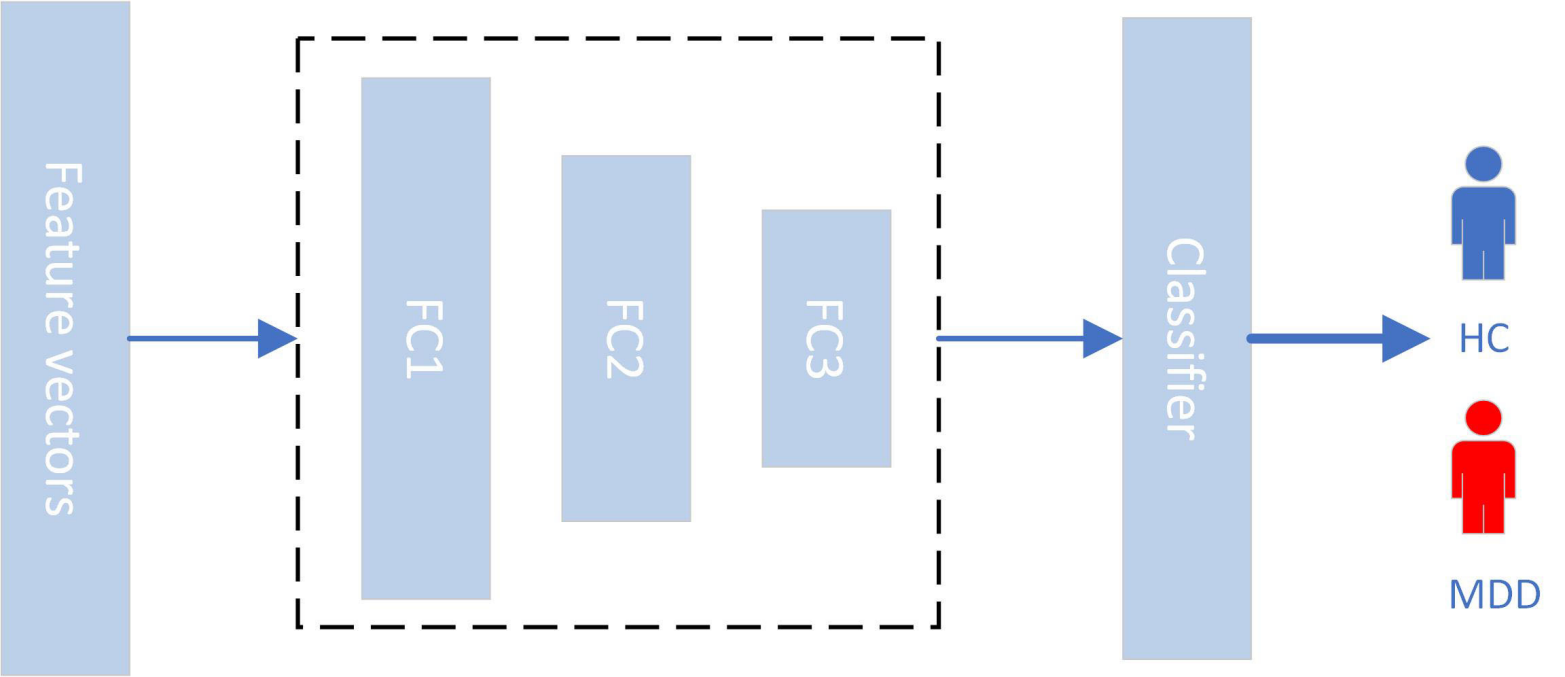
MLP model.

MLP is a fundamental model that yields high accuracy when working with small datasets. MLP is an artificial neural network consisting of multiple layers of interconnected nodes, with each node performing a simple mathematical operation. The MLP model uses the backpropagation of errors and continuously updates the weights using the gradient descent algorithm to find the minimum error value and calculate the weights of each node, as shown in **Figure 4**.

In this study, we employed a 3-layer neural network with the following parameters: (8,512), (512,1024), and (1024,1). The activation functions used were ReLU and Sigmoid. We utilized the Adam optimization method and the BCELoss as the loss function. The programming environment for this study was PyTorch version 1.13.1. Since the dataset size was small, the hardware requirements were not high, and training could be performed using a CPU.

## 3 Results

### 3.1 Classification and predication results

**Figure 5:**
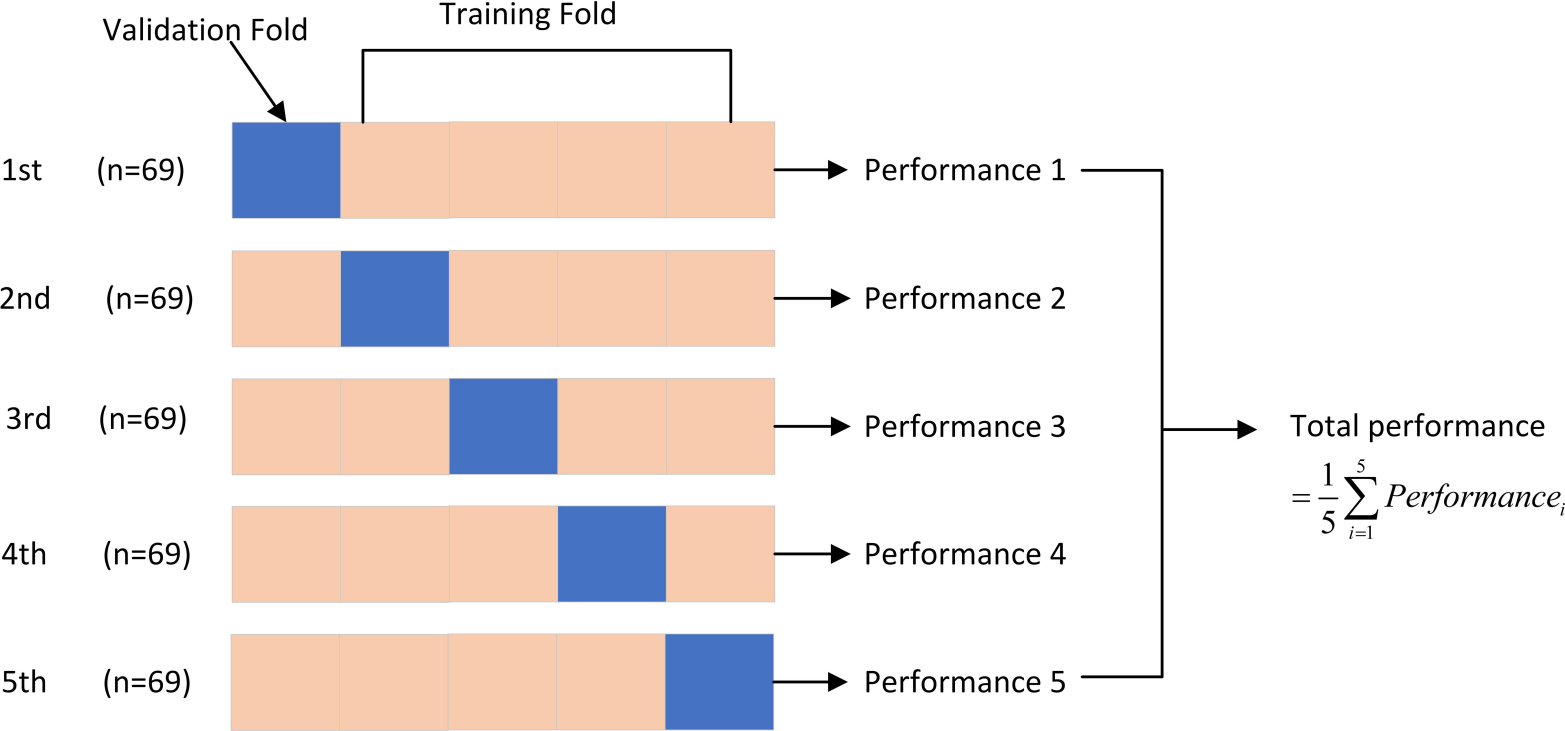
Five-fold cross-validation.

Five-fold cross-validation was performed during the classification training process, as illustrated in **Figure 5**. This process involved dividing the data into five parts: one part for validation data and the remaining four parts for training data. The final result was obtained by averaging the results of the five-fold cross-validation runs.

**Figure 6:**
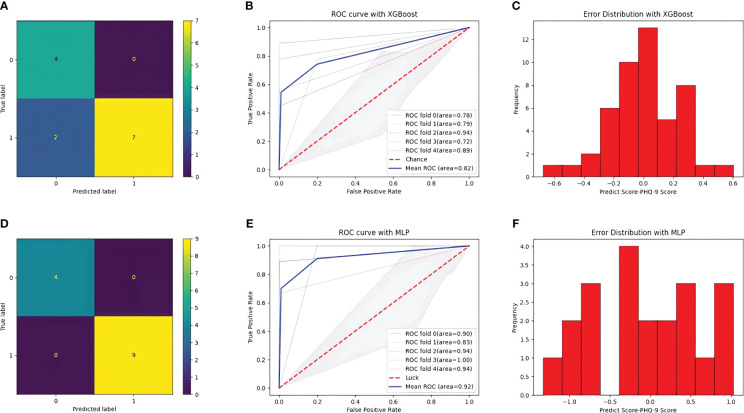
Result of the two models. **(A)** confusion matrix with XGBoost model. **(B)** ROC curve with XGBoost model. **(C)** Error distribution with XGBoost model. **(D)** confusion matrix with MLP model. **(E)** ROC curve with MLP model. **(F)** Error distribution with MLP model.

**Figure 7:**
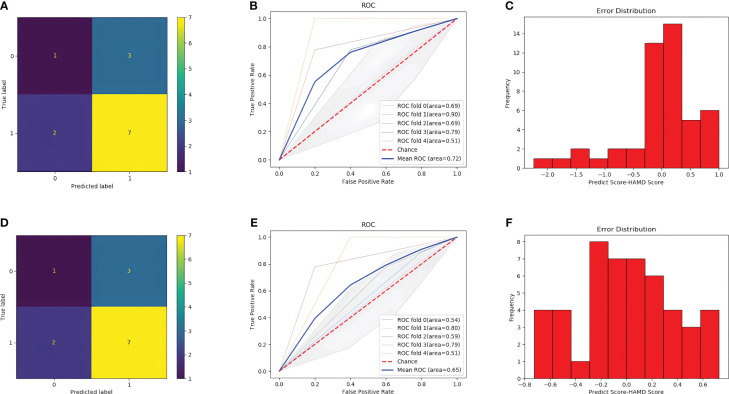
Result of the two models. **(A)** confusion matrix with SVM model. **(B)** ROC curve with SVM model. **(C)** Error distribution with SVM model. **(D)** confusion matrix with Random Forest model. **(E)** ROC curve with Random Forest model. **(F)** Error distribution with Random Forest model.

After training, the classification performance of each of the four classifiers was evaluated, and the results are presented in **Table 2**. The confusion matrix and receiver operating characteristic curve are shown in **Figures 6**, **7**, respectively. The average areas under the receiver operating characteristic curve after fivefold cross-validation were 0.86 and 0.82 for the MLP and XGBoost models, respectively. However, in terms of predicting PHQ-9 scores based on eye movement data, the XGBoost model exhibited superior performance compared with that of the MLP model. The XGBoost and MLP models predicted PHQ-9 scores with mean absolute error values ranging from -0.6 to 0.6 and from -1 to 1, respectively, indicating good predictive performance.

**Table 2:** The performance of the four classifier models.

	Fold1	Fold2	Fold3	Fold4	Fold5	Mean-Value
(A)XGBoost						
Accuaray(%)	71	79	65	79	85	76
Precision(%)	85	100	100	87	100	94
Recall(%)	67	67	73	78	78	73
F1-score(%)	75	80	65	82	88	78
AUC(%)	76	86	75	82	92	82
(B)MLP						
Accuaray(%)	85	92	85	78	92	86
Precision(%)	88	90	88	87	100	96
Recall(%)	88	100	88	77	88	91
F1-score(%)	88	94	88	82	94	92
AUC(%)	84	90	84	78	94	86
(C)SVM						
Accuaray(%)	71	92	71	78	61	74
Precision(%)	77	90	77	87	70	80
Recall(%)	77	100	77	77	77	82
F1-score(%)	77	94	77	82	73	81
AUC(%)	68	90	68	78	51	72
(D)Random Forest						
Accuracy(%)	78	64	64	85	46	67
Precision(%)	87	75	75	88	60	77
Recall(%)	77	66	66	88	66	73
F1-score(%)	82	70	70	88	63	74
AUC(%)	78	63	63	84	33	65

### 3.2 Analysis of model feature importance

**Figure 8:**
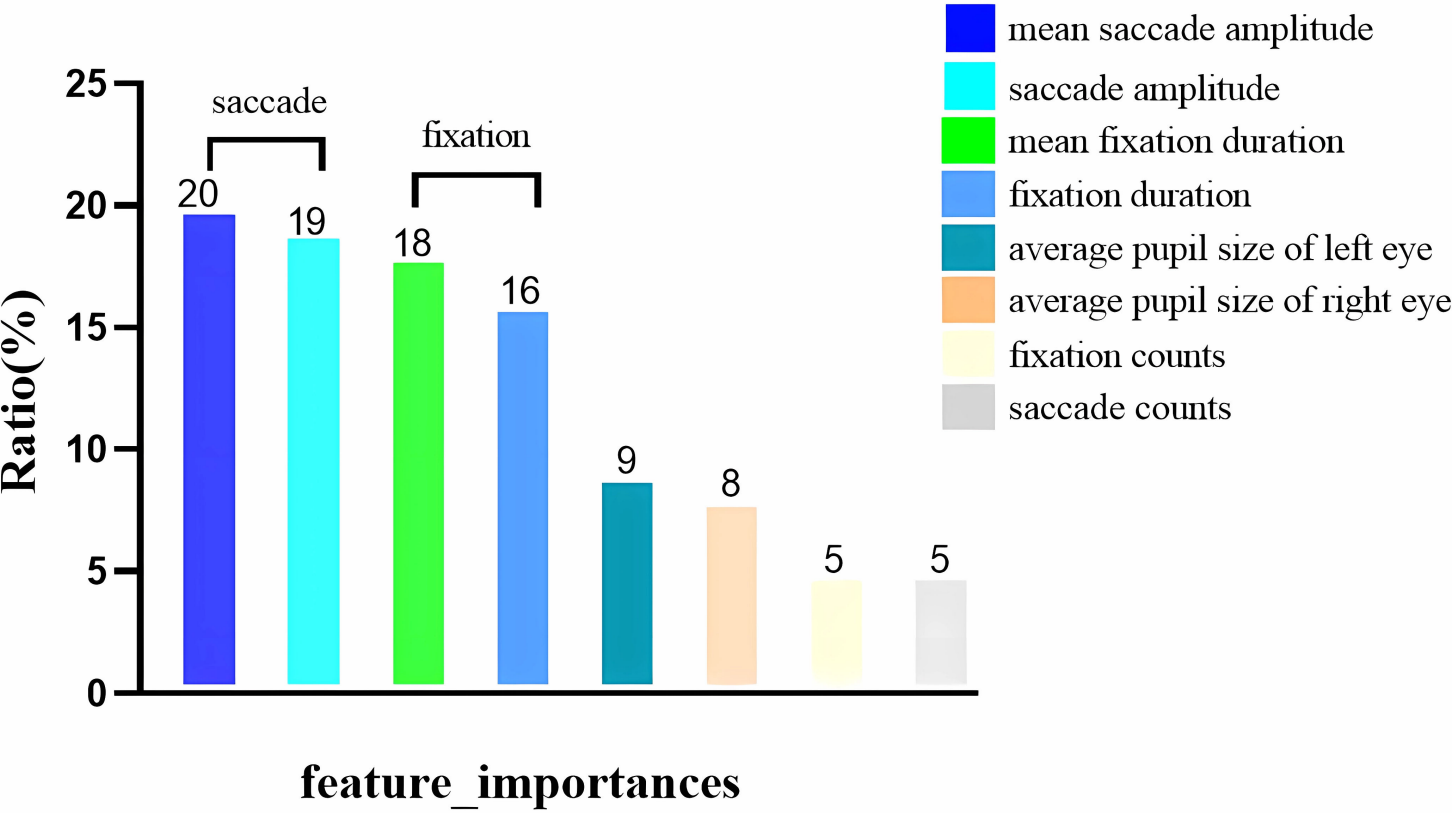
Model features importance.

The importance of each model feature was evaluated using the model’s “feature_importances_function.” The saccade and fixation indices had relatively large proportions in the model. Saccades and fixations accounted for 39% and 34% of the model’s feature importance, respectively. Together, these two indices accounted for 73% of the model’s feature importance. The feature importance of the model is illustrated in **Figure 8**, which provides a visual representation of the relative importance of each feature in the model.

### 3.3 Comparison before and after intervention

#### 3.3.1 Changes in indices

**Figure 9:**
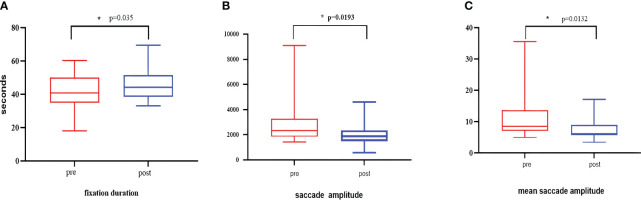
The graph compares the indicator values before and after the intervention. **(A)** Changes in fixation duration. **(B)** Changes in mean saccade amplitude. **(C)** Changes in saccade amplitude. The p-values in this case are all less than 0.01 and greater than 0.05, indicating statistical significance. They are denoted with an asterisk (*).

After five group-based CCBT sessions, significant changes were observed in the three eye movement indices. Fixation duration (p=0.035), saccade amplitude (p=0.0193), and mean saccade amplitude (p=0.0132) exhibited significant changes(as illustrated in **Figure 9**), indicating that group-based CCBT affected the eye movement patterns in individuals with depression.

#### 3.3.2 Changes in PHQ-9 scores before and after intervention

**Figure 10:**
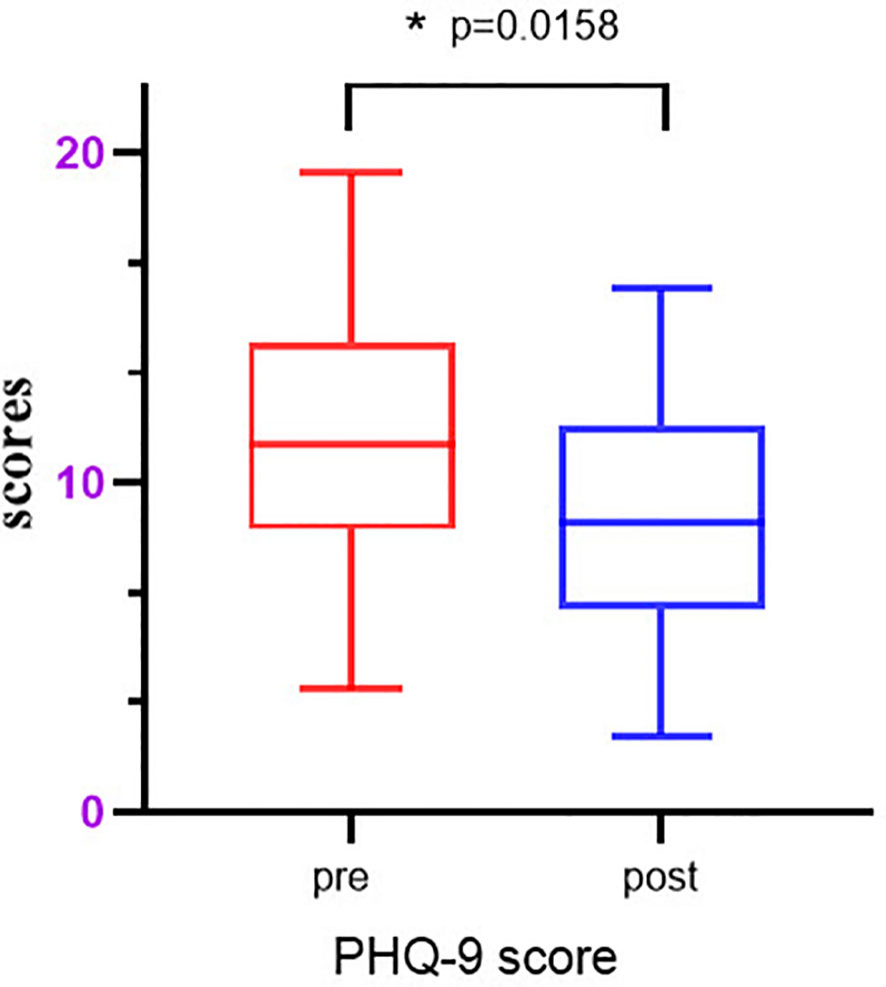
Model features importance. The p-values in this case are all less than 0.01 and greater than 0.05, indicating statistical significance. They are denoted with an asterisk (*).

The data obtained after five group-based CCBT sessions were analyzed using Prism 8 (GraphPad Software, San Diego, CA, USA). The data were found to be normally distributed, and paired t-tests were subsequently performed to assess the significance of the changes in PHQ-9 scores before and after the intervention. The results showed a significant decrease in the average PHQ-9 score from 11.57 to 7.8 after the intervention (p=0.0158) (as shown in **Figure 10**). Additionally, the depression scores for the control group remained consistent, with measurements of 12 and 11.6, indicating no change over time. These findings suggest that group-based CCBT effectively reduces depressive symptoms in individuals with depression.

## 4 Discussion

We developed a model for classifying and predicting depression that differs from those reported in prior studies, which focused solely on classification. Unlike previous studies, our model includes both classification and prediction, enabling a more comprehensive analysis of depressive symptoms.

### 4.1 Advantages of using virtual reality eye-tracking devices in research

The use of eye tracking technology in response to VR has been investigated in several studies. For example, Imaoka et al. (47) investigated the characteristics and data accuracy of eye-tracking technology using a VIVE Pro Eye device and found it to be an effective tool for evaluating eye tracking. Similarly, Alghowinem et al. (48) found that participants who used VR eye-tracking devices were more easily immersed in a scene and less likely to be disturbed by external environmental factors compared with viewing pictures.

Our findings are consistent with those of the aforementioned studies, as we also observed that using a VR eye-tracking device to watch videos provides a more realistic experience and effectively reduces attentional bias in the participants. The use of VR technology can create a more immersive and engaging experience for participants, leading to more reliable data.

### 4.2 Eye movement indices can be used to classify depression

Our study provides further evidence of the effectiveness of eye movement indices in the classification of depression, with results consistent with those of previous studies in this field. For example, Li et al. (49) analyzed eye movement data while viewing emotionally charged images from 48 individuals with depressive symptoms and 48 normal individuals. The learning machine classification method achieved the highest classification accuracy at 84.21%. In our study, the MLP model achieved a classification accuracy of 86%. These findings underscore the potential of eye movement indices as effective tools for depression classification and offer opportunities for the development of noninvasive and objective methods for diagnosing and treating depression.

Alghowinem et al. (50) used an interview method to classify eye movement data from 30 patients with depression and 30 healthy individuals and achieved an accuracy rate of 75% using a support vector machine classifier. Similarly, Zhang et al. (51) reported the highest classification accuracy at 80.1% using a random forest classifier based on data obtained when participants freely browsed pictures of emotional faces, using pupil size, gaze position, and gaze duration as classification features.

Compared with the aforementioned studies, the XGBoost and MLP models used in our study both achieved a higher classification performance. Furthermore, our study is unique in that we were able to predict PHQ-9 scores based on eye movement data, with the maximum mean absolute error ranging from -0.6 to 0.6, which is a significant improvement from that reported in previous studies.

This article utilizes four machine learning methods to establish models based on eye-tracking data for classification and prediction. The results demonstrate high levels of accuracy in both classification and prediction tasks, indicating that it can serve as an effective means for depression detection. These four machine learning methods include ensemble learning models and support vector machine models. The experiments show that both categories of models achieve significant results in utilizing eye-tracking data for depression classification and prediction, highlighting the effectiveness of these models in these tasks. Compared to the commonly used questionnaires at present, this method is quick, convenient, and objective.

### 4.3 Eye movement indices serve as biomarkers for detecting depression

Prior studies have reported that during a fixation task, patients with depression have higher fixation counts and shorter fixation durations than those of healthy controls, which is consistent with our findings. Yu et al. (52) reported similar results, highlighting the potential of eye movement data as biomarkers for the diagnosis of depression.

In our study, we observed significant differences in fixation duration (p=0.035), saccade amplitude (p=0.0193), and mean saccade amplitude (p=0.0132) after CCBT. Analysis of the predictive model parameters revealed that fixation and saccade were important influencing factors, with the importance of these two indices accounting for more than 70% of the model. These findings highlight the potential of fixation duration and saccade as biomarkers for diagnosing depression and underscore their significance in clinical diagnosis.

## 5 Conclusion

The XGBoost and MLP models were effective in classifying and predicting depression and achieved high levels of accuracy. These results suggest that machine learning techniques hold great promise for improving the diagnosis and treatment of depression, and have the potential to become valuable tools for clinicians in the future.

The analysis revealed that fixation durations and saccade counts were the two primary indices of model importance and showed significant changes following CCBT. These results suggest that fixation durations and saccade counts could serve as important biomarkers for the diagnosis of depression.

After undergoing CCBT, significant changes were observed in both eye movement indices as well as PHQ-9 scores among the participants in the control group with depressive symptoms. These results suggest that CCBT is an effective approach for managing depression.

## 6 Limitations and directions for future studies

We intend to increase the sample sizes in future studies to obtain more stable data and improve the reliability of our findings. In addition, we aim to investigate other potential biomarkers and explore the underlying mechanisms of CCBT for depression.

The number of CCBT sessions could also be increased in future studies to facilitate the collection of intermediate treatment data. Furthermore, qualitative studies could be conducted to explore the relationships between treatment duration, eye movement data, and PHQ-9 scores.

In this study, we used neutral scenes in the eye movement analysis. Future studies could also explore the use of positive and negative scenes and compare the differences in depression diagnoses.

## Data availability statement

The original contributions presented in the study are included in the article/**Supplementary Material**. Further inquiries can be directed to the corresponding author.

## Ethics statement

The studies involving humans were approved by The First Affiliated Hospital of Hainan Medical University, Haikou, China. The studies were conducted in accordance with the local legislation and institutional requirements. The participants provided their written informed consent to participate in this study.

## Author contributions

ZZ: Data curation, Formal Analysis, Methodology, Writing – original draft. LL: Conceptualization, Formal Analysis, Funding acquisition, Investigation, Writing – original draft. XL: Conceptualization, Data curation, Investigation, Writing – original draft. JC: Software, Validation, Visualization, Writing – original draft. ML: Formal Analysis, Visualization, Writing – original draft. GW: Supervision, Validation, Writing – review & editing. CX: Supervision, Validation, Writing – review & editing.
